# Supplementary material for: What is the effect of changing eligibility criteria for disability benefits on employment? A systematic review and meta-analysis of evidence from OECD countries
Source: PLoS One. 2020 Dec 1;15(12):e0242976. doi: 10.1371/journal.pone.0242976 (PMC7707516; doi:10.1371/journal.pone.0242976)
Supplement: S1 Appendix — (DOCX) [file pone.0242976.s002.docx]

Appendix S1:

Database: MEDLINE: Epub Ahead of Print, In-Process & Other Non-Indexed Citations, via Ovid MEDLINE® Daily and Ovid MEDLINE® 1946 to Present, searched 12^th^ April 2017.

--------------------------------------------------------------------------------

1 "Organisation for Economic Co-Operation and Development"/ (75)

2 "organi?ation for economic co-operation and development".ti,ab. (645)

3 "organi?ation for economic cooperation and development".ti,ab. (594)

4 OECD.ti,ab. (3149)

5 exp Australia/ (122518)

6 Australia$.ti,ab. (111765)

7 Austria/ (17785)

8 Austria$.ti,ab. (14308)

9 Belgium/ (16392)

10 Belgium.ti,ab. (12895)

11 Belgian?.ti,ab. (7427)

12 exp Canada/ (142416)

13 Canada.ti,ab. (67730)

14 Canadian?.ti,ab. (50673)

15 Chile/ (11422)

16 Chile$.ti,ab. (13740)

17 Czech Republic/ (6155)

18 Czech?.ti,ab. (9620)

19 Denmark/ (42875)

20 Denmark.ti,ab. (22492)

21 (Danes or Danish).ti,ab. (23980)

22 Estonia/ (2098)

23 Estonia$.ti,ab. (2707)

24 exp Europe/ (1270372)

25 Europe$.ti,ab. (231006)

26 Finland/ (31707)

27 Finland.ti,ab. (21345)

28 Finns.ti,ab. (1449)

29 France/ (86824)

30 France.ti,ab. (52433)

31 French.ti,ab. (59949)

32 exp Germany/ (143064)

33 German$.ti,ab. (116613)

34 Greece/ (16308)

35 Greece.ti,ab. (12128)

36 Greek?.ti,ab. (11073)

37 Hungary/ (17305)

38 Hungar$.ti,ab. (13862)

39 Iceland/ (3956)

40 Iceland$.ti,ab. (5447)

41 Ireland/ (15372)

42 Northern Ireland/ (4531)

43 Ireland.ti,ab. (16610)

44 Irish.ti,ab. (7594)

45 Israel/ (26345)

46 Israel$.ti,ab. (26906)

47 Italy/ (78352)

48 Italian?.ti,ab. (41879)

49 Italy.ti,ab. (51074)

50 Japan/ (115143)

51 Japan$.ti,ab. (180597)

52 Korea/ (16506)

53 Korea$.ti,ab. (55797)

54 Latvia/ (1119)

55 Latvia$.ti,ab. (1338)

56 Luxembourg/ (637)

57 Luxembourg$.ti,ab. (862)

58 Mexico/ (32626)

59 Mexic$.ti,ab. (51949)

60 Netherlands/ (58567)

61 Netherlands.ti,ab. (39876)

62 Dutch.ti,ab. (30732)

63 New Zealand/ (34676)

64 New Zealand$.ti,ab. (45041)

65 exp North America/ (1415726)

66 North America$.ti,ab. (44204)

67 Norway/ (33970)

68 Norway.ti,ab. (25610)

69 Norwegian?.ti,ab. (16114)

70 Poland/ (45883)

71 Poland.ti,ab. (19811)

72 Polish.ti,ab. (14465)

73 Portugal/ (10054)

74 Portugal.ti,ab. (9125)

75 Portuguese.ti,ab. (10141)

76 Slovakia/ (2350)

77 Slovak$.ti,ab. (4507)

78 Slovenia/ (2171)

79 Slovenia$.ti,ab. (3230)

80 exp South America/ (127995)

81 Spain/ (64165)

82 Spain.ti,ab. (44310)

83 Spanish.ti,ab. (41461)

84 Sweden/ (65376)

85 Sweden.ti,ab. (38445)

86 Swede?.ti,ab. (38907)

87 Swedish.ti,ab. (32152)

88 "Scandinavian and Nordic Countries"/ (4714)

89 Switzerland/ (31977)

90 Switzerland.ti,ab. (19385)

91 Swiss.ti,ab. (27605)

92 Turkey/ (29272)

93 Turkey.ti,ab. (29572)

94 Turk?.ti,ab. (2228)

95 exp United Kingdom/ (342668)

96 United Kingdom.ti,ab. (31705)

97 England/ (83223)

98 England.ti,ab. (41925)

99 English.ti,ab. (138452)

100 Scotland/ (24079)

101 Scotland.ti,ab. (14596)

102 Scottish.ti,ab. (8205)

103 Wales/ (13313)

104 Wales.ti,ab. (20375)

105 Welsh.ti,ab. (1782)

106 Great Britain.ti,ab. (6699)

107 British.ti,ab. (43578)

108 exp United States/ (1249418)

109 United States.ti,ab. (191899)

110 America$.ti,ab. (333505)

111 Developed Countries/ (19822)

112 developed countr$.ti,ab. (22653)

113 or/1-112 (4094258)

114 Fiscal Policy/ (1)

115 Policy/ (1602)

116 Social Control Policies/ (771)

117 Organizational policy/ (13586)

118 Government Programs/ (4407)

119 Government Regulations/ (19762)

120 Health Policy/ (58786)

121 Employment, Supported/ (1062)

122 Insurance, Disability/ (1366)

123 Insurance, Health/ (32523)

124 Legislation/ (1674)

125 "Legislation as Topic"/ (15921)

126 Pensions/ (3647)

127 Retirement/ (8768)

128 Sick Leave/ (4875)

129 Social Security/ (7359)

130 Workers' Compensation/ (7309)

131 or/114-130 (168990)

132 (change? or changing or eligib$ or entitlement or generosity or increas$ or introduction or reduc$ or reform$ or restrict$).ti,ab. (8216224)

133 131 and 132 (45424)

134 (benefit? adj5 (change? or changing or eligibility or entitlement or generosity or increas$ or introduction or reduc$ or reform$ or restrict$)).ti,ab. (27240)

135 (compensation adj5 (change? or changing or eligibility or entitlement or generosity or increas$ or introduction or reduc$ or reform$ or restrict$)).mp. (2945)

136 (insurance adj5 (change? or changing or eligibility or entitlement or generosity or increas$ or introduction or reduc$ or reform$ or restrict$)).ti,ab. (4559)

137 (legislat$ adj5 (change? or changing or eligibility or entitlement or generosity or increas$ or introduction or reduc$ or reform$ or restrict$)).mp. (4695)

138 (policies adj5 (change? or changing or eligibility or entitlement or generosity or increas$ or introduction or reduc$ or reform$ or restrict$)).mp. (7609)

139 (policy adj5 (change? or changing or eligibility or entitlement or generosity or increas$ or introduction or reduc$ or reform$ or restrict$)).mp. (16816)

140 (program$ adj5 (change? or changing or eligibility or entitlement or generosity or increase? or introduction or reduc$ or reform$ or restrict$)).mp. (44811)

141 (retirement? adj3 age? adj5 (change? or changing or eligibility or entitlement or generosity or increas$ or introduction or reduc$ or reform$ or restrict$)).ti,ab. (190)

142 (retirement? adj3 benefit? adj5 (change? or changing or eligibility or entitlement or generosity or increas$ or introduction or reduc$ or reform$ or restrict$)).ti,ab. (37)

143 (sickness absence? adj5 (change? or changing or eligibility or entitlement or generosity or increas$ or introduction or reduc$ or reform$ or restrict$)).ti,ab. (457)

144 (sickness benefit? adj5 (change? or changing or eligibility or entitlement or generosity or increas$ or introduction or reduc$ or reform$ or restrict$)).ti,ab. (34)

145 (social insurance adj5 (change? or changing or eligibility or entitlement or generosity or increas$ or introduction or reduc$ or reform$ or restrict$)).ti,ab. (64)

146 (wage subsid$ adj5 (change? or changing or eligibility or entitlement or generosity or increas$ or introduction or reduc$ or reform$ or restrict$)).ti,ab. (4)

147 or/134-146 (104468)

148 133 or 147 (139455)

149 (disab$ adj3 retirement?).ti,ab. (298)

150 (early adj3 (retire? or retiring or retirement?)).ti,ab. (1147)

151 (earning? adj5 (employment or job? or career? or occupation$ or profession$)).ti,ab. (498)

152 Employment/ (42077)

153 employment.ti,ab. (46706)

154 "exit from work$".ti,ab. (41)

155 health status indicators/ (22417)

156 health status/ (71404)

157 health status disparities/ (11263)

158 Income/ (25313)

159 (labo? force adj3 participat$).ti,ab. (887)

160 (labo?r market adj3 participat$).ti,ab. (200)

161 long-term disab$.ti,ab. (2192)

162 longterm disab$.ti,ab. (19)

163 (outcome? adj3 (employment or job? or career? or occupation$ or profession$)).ti,ab. (3261)

164 retirement/ (8768)

165 Return to Work/ (1287)

166 (return$ adj3 work$).ti,ab. (10406)

167 RTW.ti,ab. (728)

168 self-employ$.ti,ab. (1289)

169 short-term disab$.ti,ab. (232)

170 (stay$ adj2 work$).ti,ab. (310)

171 unemployment/ (6133)

172 (work$ adj3 participation).ti,ab. (1866)

173 Work/ (19508)

174 or/149-173 (238077)

175 113 and 148 and 174 (9088)

176 limit 175 to yr="1990 -Current" (8140)
