# Supplementary material for: What is the effect of changing eligibility criteria for disability benefits on employment? A systematic review and meta-analysis of evidence from OECD countries
Source: PLoS One. 2020 Dec 1;15(12):e0242976. doi: 10.1371/journal.pone.0242976 (PMC7707516; doi:10.1371/journal.pone.0242976)
Supplement: S2 Appendix — (DOCX) [file pone.0242976.s003.docx]

Appendix S2

| **Criteria** | **Rationale** | **Scoring** | **SCORE** |
| --- | --- | --- | --- |
| **Unit of analysis** | There were three types of analysis units used in the studies, aggregate (ecological), individual or repeated measures on the same individuals (panel). Panel data was seen as being the most robust as it allows for unmeasured confounding factors to be accounted for where these do not vary within individuals over time. Ecological studies were seen at the least robust as ecological bias can occur where aggregate data are used to make inferences about individuals.[1] | 3: Longitudinal (panel) data  2: Individual data (repeated cross section)  1: Ecological (aggregate data) |  |
| **Comparison approach** | Studies either investigate changes over time before and after an intervention or using a difference in differences approach. Studies that look at changes in the same group over time will overcome bias to a certain extent; however, the results will be at risk of being influenced by other secular trends. The more robust approach will be where a policy has changed over time for one group and this is compared with another group that is unaffected by the change (a difference in differences approach). | 3: Difference in Differences *** AFFECTED VS UNAFFECTED BEFORE/AFTER POLICY OVER TIME  1: Interrupted time series *** COMPARISON OVER TIME OF AFFECTED GROUP ONLY |  |
| **Sample selection** | Studies either use: (1) nationally recognised surveys based on random sampling, (2) non- random but representative data, for example administrative data from a scheme with universal coverage, or (3) a non-random sample not representative of the rest of the population such as administrative data from a scheme without universal coverage. | 3: Nationally recognised survey, based on random sampling OR total administrative data  2: Non-random sample that is representative (incomplete admin)  1: Non-random sample that is not representative |  |
| **Number of time points of data** | A large number of time points enables more robust analysis that better accounts for long term trends in exposed and unexposed groups. | 3: >5 time points – with at least 2 after policy start  2: 3-5 time points – with at least 2 after policy start  1: Only one time point after policy start. |  |
| **Exogeneity of policy exposure** | The potential for bias will depend on the extent to which variation in exposure to the policy change is likely to be exogenous (unlikely to be associated with confounders – nearly random) | 3: Policy variation is as good as random, un targeted roll out / arbitrary eligibility criteria.  2: Policy variation depends on administrative decision unlikely to be associated with outcomes. E.g. different jurisdictions.  1: Policy variation relates to targeting /uptake / differential adoption of policy – likely to be associated with outcomes. E.g. targeting areas with poor initial outcomes. |  |
| **Confounding** | The potential for confounding factors to bias the results will depend on:  Whether measured confounders were adequately adjusted for in the analysis (Age, Sex, Health status, Labour market conditions, wage, education or occupation.)  Whether methods were used to account for unobserved confounders (e.g. fixed effects) | 3: Most time varying confounders controlled for in analysis and unobserved time invariant confounders (using fixed effects or instrumental variable)  2: Most time varying confounders controlled for, or unobserved time invariant confounders controlled for.  1: Missing important time varying confounders controlled, and unobserved time invariant confounders not controlled for. |  |
| **Sample size / power** | The likelihood of the analysis resulting in biased estimates will also depend on the power of the study. This will depend primarily on the sample size. | 3: Priori power calculations performed indicating sufficient power / large sample size >500 observations.  2: No power calculations – sample size 100-500  1: No power calculations – sample size <100 |  |
| 1 Greenland S. Ecologic versus individual-level sources of bias in ecologic estimates of contextual health effects. International Journal of Epidemiology 2001; 30:1343-50. | | | |
