# Supplementary material for: What is the effect of changing eligibility criteria for disability benefits on employment? A systematic review and meta-analysis of evidence from OECD countries
Source: PLoS One. 2020 Dec 1;15(12):e0242976. doi: 10.1371/journal.pone.0242976 (PMC7707516; doi:10.1371/journal.pone.0242976)
Supplement: S3 Appendix — (DOCX) [file pone.0242976.s004.docx]

Appendix S3

|  | Disability prevalence, as a percentage of 20-64 population, late 1990s | Employment rate  20-64 population, late 1990s - All | Employment rate  20-64 population, late 1990s – people with disabilities |
| --- | --- | --- | --- |
| Austria | 13.0 | 68.1 | 43.4 |
| Canada | 16.1 | 74.9 | 56.3 |
| Netherlands | 18.8 | 61.9 | 39.9 |
| Spain | 11.3 | 50.5 | 22.1 |
| Sweden | 20.6 | 73.7 | 52.6 |
| UK | 18.2 | 68.6 | 38.9 |
| USA | 10.7 | 80.2 | 48.6 |
| Average | 16 | 68 | 43 |

If the pooled effect size of 1.006 was the true effect of the policy, estimated at the average employment and disability prevalence (assuming no spill-over effects) this would indicate a

(68*1.006-68)/16 = 2.6 percentage point absolute increase in the employment of people with disabilities.
