# Supplementary material for: What is the effect of changing eligibility criteria for disability benefits on employment? A systematic review and meta-analysis of evidence from OECD countries
Source: PLoS One. 2020 Dec 1;15(12):e0242976. doi: 10.1371/journal.pone.0242976 (PMC7707516; doi:10.1371/journal.pone.0242976)
Supplement: Attachment — Submitted filename: Ref_Report_PONE-D-20-19352.pdf [file pone.0242976.s005.pdf]

Referee Report for PLOS One, PONE-D-20-19352

Paper Title: "What is the effect of changing eligibility criteria for disability benefits on employment? A systematic review and meta-analysis of evidence from OECD countries"

Summary: This paper is interested in the effects of changing disability eligibility criteria on employment. Some policymakers have elected to restrict eligibility criteria in recent years to incentivize those who do not truly need the benefits to return to the labor force, but if these restrictions are not followed by an uptick in employment it is possible these restrictions have done little more than pull the safety net out from underneath individuals who do not have the ability to find or keep employment. Thus, the efficacy of these changes is an important policy concern. This paper attempts to summarize the available evidence using meta-analysis techniques, combining 17 studies from eight different OECD countries, primarily the U.S. and Canada.

Ultimately, the authors find no significant relationship between employment and changing eligibility criteria. As a result, they express concern regarding whether such policies expose individuals to poverty and put their health at risk.

Originality: Although the relationship between disability eligibility criteria and employment has been studied previously, as evidenced by the body of work this paper draws on for its meta-analyses, the notion to synthesize these different works in a rigorous and (relatively) objective framework clearly has merit. There is one other meta-analysis on this topic, which the authors here both cite and discuss. In particular, this discussion argues why the current manuscript yields new and valuable insights beyond the previous work.

Overall, the paper identifies a gap in our current knowledge that it aims to fill and demonstrates sufficient originality to merit a potential publication.

Publishability: The primary concerns that I have with the paper regard the actual implementation of the meta-analysis. As I detail in my comments below, it seems the narrative of the paper contradicts itself in some places, is less clear than it can (or should) be in others, and overall would benefit greatly from additional polish and attention to detail. I have done my best below to explain these thoughts in relation to where they came up within the current version of the manuscript.

Recommendations by Location

1. (Lines 56-57): The logic here is not necessarily true as it depends on the rate at which average age and retirement age are increasing. That's not to say it can't be true, but it's a claim that needs to be backed up especially if it is forming the core of the research's motivation.

2. (Lines 67-69): These few lines encapsulate a running concern I have with this manuscript that I'll return to throughout this report. At a fundamental level, a meta-analysis should be about combining multiple estimates of some underlying parameter of interest to increase the precision beyond what any single estimate could provide on its own. The obvious concern being pointed out here is that, for something like disability benefits, other institutional factors matter, including access to health care and other social safety net policies. As a result, it can be challenging to defend the combination of estimates across countries (here across the U.S. and Europe).

   It is perfectly reasonable to limit a meta-analysis to a specific group of countries, either because the research question being asked focuses on that group or because the best evidence comes from there. However, throughout this manuscript I see comparisons (primarily U.S. vs. other) of estimates that seem to imply to me that the authors themselves see these as two different "groups" of estimates that should not be considered as independent draws from the same underlying treatment effect distribution; these lines are the first instance of this. As a result, it is unclear to me why the authors decide to combine all of the results during their meta-analysis, ignoring these differences and (at least implicitly) assuming that such results are directly comparable.

3. (Lines 74-75): This is related to my point above. It is a good point that, although similar in absolute terms, the effects of expanded eligibility may not simply be -1 times the effects of restricted eligibility. However, in your meta-analysis you effectively treat these as the same in most cases. Your funnel plot combines them and, although you admittedly separate them in Figure 2, I do not see any discussion of why we should believe these effects mirror one another. The empirical evidence I see here seems to imply that they don't look statistically different from one another, implying that the conclusions of the previous literature are unaffected by this beyond having greater imprecision. However, given the work here is still rather imprecise, that does not yield a clear improvement.

4. (Line 81): The analysis here only includes eight countries (out of a possible 37), so this seems to be, at best, a marginal improvement. Furthermore, of those eight, three are represented by a single study. Perhaps these studies included are of a higher quality, but I do not see a compelling argument for quantity of studies as a marked improvement.

5. (Lines 84-85): Why is regional variation within a country (your example is across Canadian provinces) a limitation? I would see a group of studies using cross-region within-country variation as preferable to a series of cross-country studies.

6. (Lines 96-98): Why do we believe that these estimates across countries are comparable? You've already argued earlier (Lines 67-69) why U.S. studies should be thought of differently from other OECD countries and it appears that Barr et al. (2010) stated this explicitly in their exclusion of such studies. Why the change in stance? I think it's reasonable to be skeptical to assume that U.S./non-U.S. studies are drawing from the

same underlying treatment parameter distribution, even conditional on assuming all such estimates are unbiased and causal, so this decision requires motivation and justification.

7. (Lines 123-126): How do you handle the wide variety of age ranges included in these studies? There are a large number of factors here that may influence both the probability of being (or becoming) disabled. You say that the study must incorporate older workers, and while many of these studies focus at or near your age range of 50-65, those that do not will be biased from the effect on older workers towards the average effect for all workers. If you believe those effects are the same, you should defend that claim. If you do not believe so, you should discuss how these studies will potentially bias your results as my guess would be the inclusion of younger workers pushes you towards a null effect. Given that's your ultimate conclusion, could be a real concern.
8. (Lines 144-150): With only 17 studies in your final data set, why not apply this double-checking process to all of them?
9. (Lines 176-179): You criticized the informativeness of papers studying increased access (Lines 74-76) because that's not the direction of most current policy changes. This criticism, of course, has merit. However, here you assume that the effects are reflexive and I am unsure why you have decided to not only back off of their informativeness but make the additional assumption that the effects are 1-for-1 in the opposite direction.
10. (Line 238): Each paper only showed approximately 2 regression estimates? Or have you pared down the estimates in some other manner that you haven't mentioned? It's relatively common to restrict each study to it's main or primary conclusions, so I don't have any concerns regarding that, but an explicit acknowledgment should be included.
11. (Lines 240-242): Why were these sex-specific results excluded? You have many male- or female-only estimates in Figure 2.
12. (Lines 248-249): The figure does not show this claim. It shows that five of the results showed expanded eligibility was associated with *a statistically significant decrease* in employment. The studies in the middle may have also showed a negative association, albeit insignificant.
13. (Lines 274-275): Again, if there's a clear delineation between the U.S. and non-U.S. studies why lump them together in your meta-analysis and ignore the clear cross-country heterogeneity? I understand that statistical power is a classic limitation of this type of work, but these studies don't appear to be fundamentally comparable in this manner and, if they are, it would require discussion and justification.
14. (Lines 329-330): There's a fundamental issue here that I believe the authors need to discuss. Namely, that is the "bite" of these disability policies. It seems most studies find a treatment effect estimate that is in line with the hypothesis that restricted eligibility increases employment and relaxed eligibility decreases employment. However, most studies also find a small effect in absolute terms and this effect is most often statistically insignificant. However, the authors do not discuss the prevalence of disabilities within the broader population. To demonstrate, imagine the "true" RR of relaxing eligibility was a decrease in employment by 1 percentage point for the sample being studied. Most people will not be affected by disabilities, though. So, to calculate the effect of this policy *on disabled workers*, the estimate needs to be scaled by their prevalence in the sample studied. If 10 percent of the population were affected by this policy change, then the

effect *on them* would be 10 times as large. This is especially important here since, as the authors point out, some of these studies use the population of workers in a given country. As a result, to truly understand the effect of this policy change on affected workers the estimates need to be scaled. This also comes, of course, with the implicit assumption that there are no spillover effects as a result of the policy on non-disabled workers. This would also introduce a potential bias when using the population of workers, but my gut feeling is that this bias is secondary in nature. At minimum, this concern needs to be discussed and descriptive statistics need to be provided giving the reader the potential magnitude and scope of this concern.

15. (Figure 3): It is hard to determine the median value from this figure, but it appears to be to the right of 1.0. As with my comment above, even a small effect in absolute terms could be large when looking more closely at the group of workers actually affected.
16. (Lines 360-361): Can you reject that all of these estimates are the same? This statement can be backed up empirically but given your relatively small sample size I'm not sure what you'll find.
17. (Line 394): What was the result of your FAT? One way to really enhance the arguments put forth in this manuscript would be to provide direct, quantifiable estimates wherever possible. At present, the manuscript does this inconsistently.
18. (Lines 397-405): I must admit, I am a bit unsure what this paragraph is trying to accomplish. As I read it, it seems to me to argue against the quality of the studies included in the meta-analysis. That's not to say the points raised here are not valid, but it seems to undermine the idea of performing a meta-analysis with such studies in the first place.
19. (Lines 409-419): It could also be the case that there is an effect but its size is small when examined at the population level. As stated earlier, I think if this type of claim is going to be made (i.e. that the effects are close to zero) at the very least the upper end of the CI needs to be reported as the largest effect size the authors are willing to rule out. But this also needs to account for the bite of the policy. Perhaps the bite is quite large and so the effects do not need to be scaled a great deal but it would still need to be addressed.

Minor recommendations

1. (Throughout): The polish of the writing is quite rough throughout the manuscript and it needs a thorough proofreading.
2. (Figure 3): Figure 3 should have the standard error on the y-axis changed to the precision (1/se). That way the graph itself does not need to look any different, but the y-axis can be increasing from bottom to top rather than decreasing. The flipped axis initially confused me as I was looking at it.
3. (Lines 424-425): I'm not completely sold on making generalizations to OECD countries given that only 20% of its countries are represented here (eight out of a possible 37) and that there seems to be a great deal of heterogeneity across the U.S./non-U.S. studies.

References

Barr, B., Clayton, S., Whitehead, M., Thielen, K., Burström, B., Nylén, L., & Dahl, E. (2010). To what extent have relaxed eligibility requirements and increased generosity of disability benefits acted as disincentives for employment? A systematic review of evidence from countries with well-developed welfare systems. *Journal of Epidemiology & Community Health, 64*(12), 1106-1114.
