# Supplementary material for: What is the effect of changing eligibility criteria for disability benefits on employment? A systematic review and meta-analysis of evidence from OECD countries
Source: PLoS One. 2020 Dec 1;15(12):e0242976. doi: 10.1371/journal.pone.0242976 (PMC7707516; doi:10.1371/journal.pone.0242976)
Supplement: Attachment — Submitted filename: RefereeReport_DisabilityBenefits_Plosone2020.pdf [file pone.0242976.s006.pdf]

Referee Report for: What is the effect of changing eligibility criteria for disability benefits on employment? A systematic review and meta-analysis of evidence from OECD countries

Manuscript Number: PONE-D-20-19352

Summary:

The paper reviews all the articles published in the literature between 1990 to June 2018 that focus on the evaluation of policies that modify either the eligibility requirements of disability benefits or the income replacement rate of those benefits and that analyze the effects of those policy changes on one outcome; the employment probabilities of disabled individuals. The inclusion criteria also specifies that the focus has to be the countries of the OECD. Therefore, papers that do not study a policy change or that are centered around the effects on other outcomes (such as the inflow into the disability system) or that apply the analyses on non-OECD countries are excluded from the review.

Following these criteria, the authors end up with seventeen studies from seven countries which include 36 results from different policies.

The results of the review point towards the lack of any significant employment effects of changes in the eligibility criteria for disability benefits, which include both studies focusing on restrictions as well as on expansions of the eligibility conditions. Therefore, the authors conclude that restricting the eligibility criteria for disability benefits will not lead to increased employment while leaving an important proportion of disabled individuals without benefits and increasing their poverty risk.

The paper provides a good review of a very specific topic and does a very good job in the selection of the papers. Furthermore, the authors also make an effort to homogenize the findings across papers from different countries and focusing on different policies so as to make them comparable and extract some common conclusions. However, as the focus of the review is rather narrow, I think that the paper can make a stronger point in the concluding section trying to incorporate some other important papers looking at similar although somewhat different policies. This exercise will expand the picture given by the review and will make it more useful from a policy perspective. Furthermore, some elements are missing in the explanation of the homogenization process of the different results in the reviewed papers. In what follows, I expand on the points that I think could help strengthen the paper.

I enumerate and explain these issues in more detail in what follows:

1. Inclusion criteria: I believe that the authors should explicitly mention whether they restrict their focus on the permanent disability system or whether they also include papers looking at the temporary disability system. Similarly, are they only focusing their search on public disability benefits or also on employer-provided benefits?
2. When homogenizing the results of the different papers reviewed, the authors don't explain how they value changes in replacement rates. It is easy to interpret the homogenization when the policy is to restrict or to expand access to the benefits but how do they measure it if, for example, the policy change is a reduction in the replacement rate by, say 10%? It is obvious that it will not have the same effect a reduction by 10% than a reduction by 60% and this should be somehow incorporated in the homogenization process of the results.
3. The review includes policy changes form the 1980's, 1990's and the 2000's and the labor market situation across all these years is clearly different in OECD countries. Thus, the results obtained in the papers analyzed may be clearly dependent on the labor market situation at the time of the reform. Therefore, this may explain some of the differences in findings from similar policies which were applied in different decades. There is evidence in the literature (for several countries) that labour market conditions determine the inflow into disability benefits and that, therefore, individuals entering the DI system in good or bad economic times might be different in several characteristics, including the probability of working. For example, for the USA, the paper by Maestas et al. (2015) shows that the increased inflow into DI during recessions is driven by "conditional applicants; that is, workers with health impairments who would prefer to remain in the labor force, but who apply for SSDI benefits if they lost their present job (Autor and Duggan, 2003)". In fact, the paper by Cow and Rutledge (2013) finds that applicants during bad economic times have higher past earnings and more recent work experience so that they might be more likely to go back to work if an employment promotion policy is introduced during recessionary periods. In a similar vein, the paper by Koning and Vethaak 2019 use administrative data for The Netherlands from 1999 to 2013 to show that the observed decrease in employment rates of disability applicants during the period studied is mainly attributable to changes in the composition of applicants with increasing shares of workers without permanent contracts in the year before the application.

   Therefore, in view of this evidence, I would suggest that these elements should be discussed in the paper and that the authors should show their results differentiated by decade or some temporal division that takes into account booming and recessionary

periods (either in the main results section or in a separate section such as a robustness check).

4. The baseline replacement rate in the different countries may also be a significant explanatory variable to understand why some studies find effects while others don't. It is not the same to have a 100% replacement rate (with respect to previous wages) than to have 70%; the incentives to work may not be equally strong in both cases.
5. In the concluding section, the results of these policies may not be the same for countries that have experienced an increase in the inflow rates into disability benefits (like The Netherlands, or the USA) than in countries that have kept a relatively flat inflow rate (like Spain) as the level of disability of beneficiaries and the underlying health condition may be different in those two types of countries.
6. Also in the concluding section, I would suggest to include information on some policies (different from the ones reviewed in the paper) that have proven to work in promoting the employment of disabled workers, such as gradual return-to-work policies or making employers responsible for paying sickness benefits. The case of The Netherlands provides several examples of the success of some of these reforms. For example, the paper by Kools and Koning (2019) shows that graded return-to-work policies accelerate work resumption, which is achieved at a faster pace (except for individuals with mental health problems), and have also the power of speeding up the recovery process. Similarly, the paper by Hullegie and Koning 2018 proves that, a reform that both makes employers responsible for paying sickness benefits as well as strengthens their monitoring requirements, stimulates preventive and reintegration activities by firms and increases employment probabilities of workers after they experience a health shock.

   Thus, some activation policies may work both in terms of employment and recovery outcomes and might help restore the public budget by reducing the amount of disability insurance benefits paid by the government.

REFERENCES;

Maestas, N., Mullen, K.J. and Strand, A. 2015. "Disability Insurance and the Great Recession", American Economic Reviews, 105(5): 177-182.

Autor, D. and Duggan M. 2003. "The rise in the disability rolls and the decline in unemployment". Quarterly Journal of Economics, 118 (1); 157-206.

Coe, N.B and Rutledge MS. 2013. “How does the composition of disability insurance applicants change across business cycles?”. Center for Retirement Research at Boston College Working Paper 2013-5.

Koning, P. and Vethaak, H. 2019. “Decomposing employment trends of disabled workers”. IZA Discussion Papers No.12775.

Kools, L and Koning, P. 2019. “Graded return-to-work as a stepping stone to full work resumption”. Journal of health economics, 65; 189-209.

Hullegie, P. and Koning, P 2018. “How disability insurance reforms change the consequences of health shocks on income and employment”. Journal of Health Economics 62, 134-146.
