# Supplementary material for: What is the effect of changing eligibility criteria for disability benefits on employment? A systematic review and meta-analysis of evidence from OECD countries
Source: PLoS One. 2020 Dec 1;15(12):e0242976. doi: 10.1371/journal.pone.0242976 (PMC7707516; doi:10.1371/journal.pone.0242976)
Supplement: Attachment — Submitted filename: Response to reviewers.docx [file pone.0242976.s007.docx]

Dear Konstantinos

**RE: Response to peer review for PONE-D-20-19352**

Thank you for the peer-review reports. We have considered them thoroughly and have given a response to specific points in the reports below. We have also noted there was a coding error for Gruber (2000) when converting the paper result to a relative rate, this has been amended and results have been updated accordingly. With regard to the specific questions you have raised:

1. **The comparability of the studies across set of countries (e.g. US vs. non-US), raised by both reviewers R1 and R2, as well as the comparability over time given that policy interventions occurred under different economic conditions (R2). Presenting evidence separately by group of countries and over time would strengthen this part of the analysis.**

*We have added table 3 to the results section, which include meta-regressions by separate stratifications of results (by age, sex, USA vs. non-USA and decade of policy reform). We have also expanded the discussion to specifically comment on the issues raised by the comparisons,*

2. **Reflect on the assumption of reversibility of policy effects (R1) as well as consider the role of the magnitude of the policy change and the pre-reform differences in benefits generosity (R2).**

*We have added these issues to the limitations and included a stratification of meta-regression based on whether policy change was an expansion or restriction of disability benefits. Additionally, our narrative analysis already considers these policy approaches separately.*

3. **Clarify several choices such excluding sex-specific results and age restrictions (R1).**

*This has been explained in the manuscript now. See point 11 in response to reviewer 1.*

4. **Consider the detailed comments by R1 on the consistency of the arguments in several parts of the manuscript.**

*See responses to reviewer 1.*

5. **Consider extending the set of policies as suggested by R2**

*We have added a short section to the discussion discussing the need to consider other active labour market policies to improve the employment of people with disabilities. We feel a more detailed examination of this is beyond the scope of our paper. We explain in the paper that we were not able to focus on changes in replacement rates in detail because there were very few studies that specifically investigated this and we have added this to the limitations.*

The table below shows the specific response to reviewer comments:

| Reviewer Comment | Response |
| --- | --- |
| Reviewer 1 | |
| 1. (Lines 56-57): The logic here is not necessarily true as it depends on the rate at which average age and retirement age are increasing. That’s not to say it can’t be true, but it’s a claim that needs to be backed up especially if it is forming the core of the research’s motivation. | We agree that this is not necessarily true. Really the main issue is increased retirement age – as this will mean older age groups will need to stay in work, and these older age groups are more likely to be disabled. We have changed this sentence to reflect this point.  (55-57). |
| 2. (Lines 67-69): These few lines encapsulate a running concern I have with this manuscript that I’ll return to throughout this report. At a fundamental level, a meta-analysis should be about combining multiple estimates of some underlying parameter of interest to increase the precision beyond what any single estimate could provide on its own. The obvious concern being pointed out here is that, for something like disability benefits, other institutional factors matter, including access to health care and other social safety net policies. As a result, it can be challenging to defend the combination of estimates across countries (here across the U.S. and Europe). It is perfectly reasonable to limit a meta-analysis to a specific group of countries, either because the research question being asked focuses on that group or because the best evidence comes from there. However, throughout this manuscript I see comparisons (primarily U.S. vs. other) of estimates that seem to imply to me that the authors themselves see these as two different “groups” of estimates that should not be considered as independent draws from the same underlying treatment effect distribution; these lines are the first instance of this. As a result, it is unclear to me why the authors decide to combine all of the results during their meta-analysis, ignoring these differences and (at least implicitly) assuming that such results are directly comparable. | We thank the reviewer for their comment which raises an important concern that we have had in this and our previous review about heterogeneity of effects between policy contexts. This is a point we can make clearer and agree that the meta-analysis could be used to more effectively explore this heterogeneity – particularly between the US and non-US countries. We have now clarified this in the introduction and have included added meta-regression analysis of the results by a number of stratifications to explore this heterogeneity, including; USA and other countries, sex specific, limited to older average age populations, quality and whether the policy change is an expansion or restriction. We feel it is important to include these different analyses, as it demonstrates that the result remain broadly unchanged (with the exception of the high-quality stratification). We agree there are limitations this is discussed in the paper (508-525). |
| 3. (Lines 74-75): This is related to my point above. It is a good point that, although similar in absolute terms, the effects of expanded eligibility may not simply be -1 times the effects of restricted eligibility. However, in your meta-analysis you effectively treat these as the same in most cases. Your funnel plot combines them and, although you admittedly separate them in Figure 2, I do not see any discussion of why we should believe these effects mirror one another. The empirical evidence I see here seems to imply that they don’t look statistically different from one another, implying that the conclusions of the previous literature are unaffected by this beyond having greater imprecision. However, given the work here is still rather imprecise, that does not yield a clear improvement. | This is a good point. We have reorganised our methods slightly to forefront the investigation of heterogeneity of effects initially, in particular whether it is reasonable make this assumption about expanding and restricting reforms. This then provides some justification for the combining these in further meta-regression and pooling analysis across stratified groups. As the reviewer points out this is still quite imprecise – and we discuss this further in the limitations section |
| 4. (Line 81): The analysis here only includes eight countries (out of a possible 37), so this seems to be, at best, a marginal improvement. Furthermore, of those eight, three are represented by a single study. Perhaps these studies included are of a higher quality, but I do not see a compelling argument for quantity of studies as a marked improvement. | We agree that this is only a marginal improvement, but it highlights an important point about the evidence base. The number of countries in the previous review was limited *in the study design* to 5 countries – it was not possible from that study to identify the full distribution of evidence across countries. Our inclusion criteria has been expanded to 36 countries, the fact that we only found evidence from 7 countries reflects a limitation of the evidence base. We discuss this further in the limitations and highlight the need for more studies and the limitations of generalisability (483-486). |
| 5. (Lines 84-85): Why is regional variation within a country (your example is across Canadian provinces) a limitation? I would see a group of studies using cross-region within country variation as preferable to a series of cross-country studies. | We have clarified in the introduction – what we meant was that a limitation of previous studies was that some just analysed cross sectional differences between jurisdiction at single time point -and not specifically analysing changes over time following a change in policy in these jurisdictions (e.g. through a DiD approach for example). We have made this distinction clearer. |
| 6. (Lines 96-98): Why do we believe that these estimates across countries are comparable? You’ve already argued earlier (Lines 67-69) why U.S. studies should be thought of differently from other OECD countries and it appears that Barr et al. (2010) stated this explicitly in their exclusion of such studies. Why the change in stance? I think it’s reasonable to be skeptical to assume that U.S./non-U.S. studies are drawing from the same underlying treatment parameter distribution, even conditional on assuming all such estimates are unbiased and causal, so this decision requires motivation and justification. | We agree, it is reasonable to be sceptical that U.S./non-U.S. studies are drawing from the same underlying treatment parameter distribution. We have now reorganised the methods and results to reflect this, exploring these differences. We then discuss further our tentative conclusions that we don’t find evidence to support differences in effect size between these two groups of countries. |
| 7. (Lines 123-126): How do you handle the wide variety of age ranges included in these studies? There are a large number of factors here that may influence both the probability of being (or becoming) disabled. You say that the study must incorporate older workers, and while many of these studies focus at or near your age range of 50-65, those that do not will be biased from the effect on older workers towards the average effect for all workers. If you believe those effects are the same, you should defend that claim. If you do not believe so, you should discuss how these studies will potentially bias your results as my guess would be the inclusion of younger workers pushes you towards a null effect. Given that’s your ultimate conclusion, could be a real concern. | We have focused on studies that include older workers. However, as the reviewer points out there are a number of studies that also include younger workers and these will be biased from the effect on older workers towards the average effect for all workers.  To investigate this further have conducted stratified meta-regression comparing the effect size between studies only including older workers and those that included all workers. This suggests that there may have been slightly larger effects in studies that were limited to older age groups, the effect size limited to those studies is larger, although this is still not quite statistically significant at the 5% level (p=0.095). |
| 8. (Lines 144-150): With only 17 studies in your final data set, why not apply this doublechecking process to all of them? | Sorry, this was an error on our part, this was actually done and has been amended in the text |
| 9. (Lines 176-179): You criticized the informativeness of papers studying increased access (Lines 74-76) because that’s not the direction of most current policy changes. This criticism, of course, has merit. However, here you assume that the effects are reflexive and I am unsure why you have decided to not only back off of their informativeness but make the additional assumption that the effects are 1-for-1 in the opposite direction. | See point 3 |
| 10. (Line 238): Each paper only showed approximately 2 regression estimates? Or have you pared down the estimates in some other manner that you haven’t mentioned? It’s relatively common to restrict each study to it’s main or primary conclusions, so I don’t have any concerns regarding that, but an explicit acknowledgment should be included. | We have added text to the methods to explaining that for papers that reported results from multiple regression models (e.g. controlling for different covariates) we selected the results which had been adjusted for the highest number of appropriate covariates. |
| 11. (Lines 240-242): Why were these sex-specific results excluded? You have many male- or female-only estimates in Figure 2. | We excluded the sex specific results where pooled male and female results were available, as including both the sex specific results and the pooled analysis would have effectively meant duplicating the results from one study – i.e. there would have been 6 results just from this one study which we felt would give underweight to this one study in the visualisation. |
| 12. (Lines 248-249): The figure does not show this claim. It shows that five of the results showed expanded eligibility was associated with a statistically significant decrease in employment. The studies in the middle may have also showed a negative association, albeit insignificant. | This is a fair point – we have reworded this to reflect the data correctly to refer to significant results. The non-significant results were fairly evenly distributed either side of the null. |
| 13. (Lines 274-275): Again, if there’s a clear delineation between the U.S. and non-U.S. studies why lump them together in your meta-analysis and ignore the clear cross-country heterogeneity? I understand that statistical power is a classic limitation of this type of work, but these studies don’t appear to be fundamentally comparable in this manner and, if they are, it would require discussion and justification. | See point 2, 3 and 6 above. |
| 14. (Lines 329-330): There’s a fundamental issue here that I believe the authors need to discuss. Namely, that is the “bite” of these disability policies. It seems most studies find a treatment effect estimate that is in line with the hypothesis that restricted eligibility increases employment and relaxed eligibility decreases employment. However, most studies also find a small effect in absolute terms and this effect is most often statistically insignificant. However, the authors do not discuss the prevalence of disabilities within the broader population. To demonstrate, imagine the “true” RR of relaxing eligibility was a decrease in employment by 1 percentage point for the sample being studied. Most people will not be affected by disabilities, though. So, to calculate the effect of this policy on disabled workers, the estimate needs to be scaled by their prevalence in the sample studied. If 10 percent of the population were affected by this policy change, then the effect on them would be 10 times as large. This is especially important here since, as the authors point out, some of these studies use the population of workers in a given country. As a result, to truly understand the effect of this policy change on affected workers the estimates need to be scaled. This also comes, of course, with the implicit assumption that there are no spillover effects as a result of the policy on non-disabled workers. This would also introduce a potential bias when using the population of workers, but my gut feeling is that this bias is secondary in nature. At minimum, this concern needs to be discussed and descriptive statistics need to be provided giving the reader the potential magnitude and scope of this concern. | This is a fair point and we have added this to the discussion to explain the results in this context, we have also added descriptive statistics on disability prevalence of disabilities in OECD countries (appendix S3). One of the issues of studies of this sort as the reviewer highlights, is a lack of precision, particularly when based on whole population data. This can be improved by focusing specifically on the population affected by the policy. |
| 15. (Figure 3): It is hard to determine the median value from this figure, but it appears to be to the right of 1.0. As with my comment above, even a small effect in absolute terms could be large when looking more closely at the group of workers actually affected. | If we have understood the reviewer correctly, they are asking for the median effect size to be included in the funnel plot. We think a better measure of the average effect size would be the pooled effect from the meta-analysis – i.e. 1.006 which is already in the results, which as the reviewer says is to the right of 1.00. We agree that this needs to be interpreted in the context of the group of workers actually affected. We have added to the discussion highlighting this. |
| 16. 16. (Lines 360-361): Can you reject that all of these estimates are the same? This statement can be backed up empirically but given your relatively small sample size I’m not sure what you’ll find. | We have added the results of the meta regression to table 3 testing whether there are significant differences between study groups and sort to simplify this section limited it to a discussion of statements that can be backed up empirically. However, given the small number of studies and level of heterogeneity, we can only make cautious inferences, we discuss these limitations in the discussion. |
| 17. (Line 394): What was the result of your FAT? One way to really enhance the arguments put forth in this manuscript would be to provide direct, quantifiable estimates wherever possible. At present, the manuscript does this inconsistently. | We have included the results of the Egger’s test (399-400). |
| 18. (Lines 397-405): I must admit, I am a bit unsure what this paragraph is trying to accomplish. As I read it, it seems to me to argue against the quality of the studies included in the meta-analysis. That’s not to say the points raised here are not valid, but it seems to undermine the idea of performing a meta-analysis with such studies in the first place. | We have rephrased this slightly to clarify. We feel it is necessary to clarify the issues with the underlying studies that may influence overall findings  Firstly, there are issues with precision and power and we have added discussion of the good point the reviewer has made policies evaluated across a whole population may have small effects at the population level even if the effect were large in the target group. When taken as a whole, we feel this justifies why meta-analysis is performed but why caution is needed (433-480). |
| 19. (Lines 409-419): It could also be the case that there is an effect but its size is small when examined at the population level. As stated earlier, I think if this type of claim is going to be made (i.e. that the effects are close to zero) at the very least the upper end of the CI needs to be reported as the largest effect size the authors are willing to rule out. But this also needs to account for the bite of the policy. Perhaps the bite is quite large and so the effects do not need to be scaled a great deal but it would still need to be addressed. | We have added to the discussion a section on this. Indicating what the pooled effect size would be on the population with disabilities, assuming the average employment rate and disability prevalence across the study countries (419-431). |
| 1. (Throughout): The polish of the writing is quite rough throughout the manuscript and it needs a thorough proofreading. | Thank you – we have been through the manuscript in detail to improve readability and proof read |
| 2. (Figure 3): Figure 3 should have the standard error on the y-axis changed to the precision (1/se). That way the graph itself does not need to look any different, but the y-axis can be increasing from bottom to top rather than decreasing. The flipped axis initially confused me as I was looking at it. | Thank you, this funnel plot has been updated, and the y-axis has been converted to (1-SE) |
| 3. (Lines 424-425): I’m not completely sold on making generalizations to OECD countries given that only 20% of its countries are represented here (eight out of a possible 37) and that there seems to be a great deal of heterogeneity across the U.S./non-U.S. studies. | We have added the limitations of generalisability |
| Reviewer 2 | |
| The paper provides a good review of a very specific topic and does a very good job in the selection of the papers. Furthermore, the authors also make an effort to homogenize the findings across papers from different countries and focusing on different policies so as to make them comparable and extract some common conclusions. | We thank the reviewer for their positive comment. |
| 1. Inclusion criteria: I believe that the authors should explicitly mention whether they restrict their focus on the permanent disability system or whether they also include papers looking at the temporary disability system. Similarly, are they only focusing their search on public disability benefits or also on employer-provided benefits? | We have added clarification of this to inclusion criteria in table 1. Change to the temporary sickness/disability benefits were excluded. |
| 2. When homogenizing the results of the different papers reviewed, the authors don’t explain how they value changes in replacement rates. It is easy to interpret the homogenization when the policy is to restrict or to expand access to the benefits but how do they measure it if, for example, the policy change is a reduction in the replacement rate by, say 10%? It is obvious that it will not have the same effect a reduction by 10% than a reduction by 60% and this should be somehow incorporated in the homogenization process of the results | Of the 18 papers that were included in the review 17 evaluated changes in eligibility criteria, 5 of these reforms also included changes in replacement rates, and only 1 study investigated change in replacement rates alone. It was not possible therefore with only 5 studies to investigate whether the policy effect differed between these studies based on the amount the replacement rates changed. We have clarified this in the results and highlight the issue in the discussion (427-436). |
| 3. The review includes policy changes form the 1980’s, 1990’s and the 2000’s and the labor market situation across all these years is clearly different in OECD countries. Thus, the results obtained in the papers analyzed may be clearly dependent on the labor market situation at the time of the reform. Therefore, this may explain some of the differences in findings from similar policies which were applied in different decades. There is evidence in the literature (for several countries) that labour market conditions determine the inflow into disability benefits and that, therefore, individuals entering the DI system in good or bad economic times might be different in several characteristics, including the probability of working. For example, for the USA, the paper by Maestas et al. (2015) shows that the increased inflow into DI during recessions is driven by “conditional applicants; that is, workers with health impairments who would prefer to remain in the labor force, but who apply for SSDI benefits if they lost their present job (Autor and Duggan, 2003)”.   In fact, the paper by Cow and Rutledge (2013) finds that applicants during bad economic times have higher past earnings and more recent work experience so that they might be more likely to go back to work if an employment promotion policy is introduced during recessionary periods. In a similar vein, the paper by Koning and Vethaak 2019 use administrative data for The Netherlands from 1999 to 2013 to show that the observed decrease in employment rates of disability applicants during the period studied is mainly attributable to changes in the composition of applicants with increasing shares of workers without permanent contracts in the year before the application. Therefore, in view of this evidence, I would suggest that these elements should be discussed in the paper and that the authors should show their results differentiated by decade or some temporal division that takes into account booming and recessionary periods (either in the main results section or in a separate section such as a robustness check). | The reviewer makes some important points about how local labour market conditions could modify the effects of the policies reviewed. We have added decade of policy change to our meta-regression stratification (table 3) and expanded the discussion focussing on the potential effects of the underlying labour market conditions to discuss the issue raised in this point (457-469). |
| 4. The baseline replacement rate in the different countries may also be a significant explanatory variable to understand why some studies find effects while others don’t. It is not the same to have a 100% replacement rate (with respect to previous wages) than to have 70%; the incentives to work may not be equally strong in both cases. | As highlighted above – only a few of the studies evaluated policies that changes replacement rate – by far the majority involved primarily changes in eligibility criteria. We were not therefore able to analyse the impact of different baseline replacement rate levels. |
| 5. In the concluding section, the results of these policies may not be the same for countries that have experienced an increase in the inflow rates into disability benefits (like The Netherlands, or the USA) than in countries that have kept a relatively flat inflow rate (like Spain) as the level of disability of beneficiaries and the underlying health condition may be different in those two types of countries. | We agree that there are a number of contextual factors that may influence effectiveness, the composition of the population out of work with a disability is definitely an important one. Unfortunately, it is not really possible to investigate this in this review. We have added further to the discussion on this important point. |
| 6. Also in the concluding section, I would suggest to include information on some policies (different from the ones reviewed in the paper) that have proven to work in promoting the employment of disabled workers, such as gradual return-to-work policies or making employers responsible for paying sickness benefits. The case of The Netherlands provides several examples of the success of some of these reforms. For example, the paper by Kools and Koning (2019) shows that graded return-to-work policies accelerate work resumption, which is achieved at a faster pace (except for individuals with mental health problems), and have also the power of speeding up the recovery process. Similarly, the paper by Hullegie and Koning 2018 proves that, a reform that both makes employers responsible for paying sickness benefits as well as strengthens their monitoring requirements, stimulates preventive and reintegration activities by firms and increases employment probabilities of workers after they experience a health shock.   Thus, some activation policies may work both in terms of employment and recovery outcomes and might help restore the public budget by reducing the amount of disability insurance benefits paid by the government. | We have added a short section to the discussion discussing the need to consider other active labour market policies to improve the employment of people with disabilities. We feel a more detailed examination of this is beyond the scope of our paper (551-561). |

We hope our responses answer the points raised and we welcome any further comments. We have also uploaded updated figure (2, 3 and 4) and a new appendix S3.

Kind Regards

Dr Philip McHale

Clinical Lecturer in Public Health and Policy, University of Liverpool
